# Iatrogenic Superior Vena Cava Syndrome With Extensive Thromboembolism Associated With Chronic Indwelling Catheterization

**DOI:** 10.7759/cureus.78179

**Published:** 2025-01-29

**Authors:** Xiaojin Song, Jordan Whittles, Xuan Ding

**Affiliations:** 1 Internal Medicine, AdventHealth Orlando, Orlando, USA; 2 Medicine, Loma Linda University School of Medicine, Loma Linda, USA; 3 General Internal Medicine, Mayo Clinic, Jacksonville, USA

**Keywords:** catheter-related thrombosis, extensive thromboembolism, iatrogenic, subcutaneous venous access devices, superior vena cava syndrome

## Abstract

Superior vena cava syndrome (SVCS) results from the partial or complete obstruction of blood flow through the superior vena cava (SVC), which comprises a broad clinical spectrum ranging from asymptomatic cases to rare life-threatening emergencies with upper airway obstruction and increased intracranial pressure. Iatrogenic SVCS is not uncommon given the increasing utilization of intravascular devices in the past decades, suggesting that wider knowledge of the indications for semipermanent venous access and consistent monitoring are warranted in our clinical practice in order to avoid unfavorable outcomes. Here, we present a case of SVCS with respiratory distress due to extensive thromboembolism associated with a chronic indwelling intravenous (IV) catheter.

## Introduction

Superior vena cava syndrome (SVCS) results from any condition that leads to the partial or complete obstruction of blood flow through the superior vena cava (SVC), which impedes drainage from the head, neck, and upper extremities. SVCS can be roughly divided into two categories: malignancy related and benign or non-malignant caused. Benign or non-malignant causes of SVCS now comprise at least 40% of cases [1,2]. Several predisposing factors have been identified for non-malignant SVCS, including central venous devices, arteriovenous hemodialysis access, venous thoracic outlet syndromes, mediastinal fibrosis, hormonal therapy, and prothrombotic states [2].

Central venous catheters allow the delivery of medications and nutritional support that cannot be given safely through peripheral venous catheters and also allow the measurement of hemodynamic variables that cannot be measured accurately by noninvasive means. The past few decades have witnessed a dramatic increase in the use of semipermanent central venous access devices, both in and out of hospital. Unfortunately, this could be associated with long-term complications including venous thrombosis events. Here, we present a case of iatrogenic SVCS with extensive thromboembolism associated with chronic indwelling catheterization.

## Case presentation

**Figure 1:**
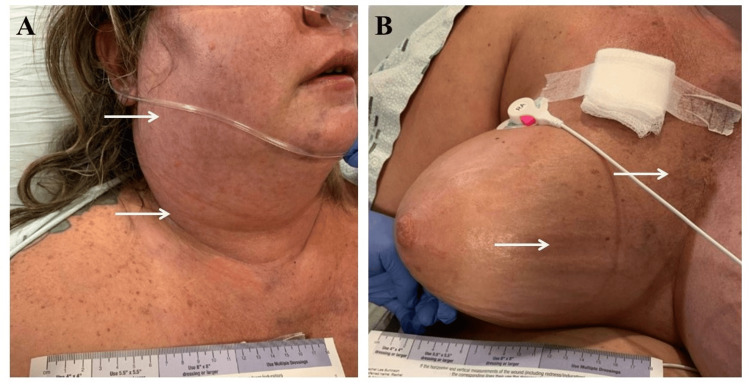
Photographs of the head and upper chest of a 42-year-old woman with catheter-associated SVCS showing plethora and prominent superficial venous pattern on (A) the face and neck and (B) the upper chest and breast. SVCS: superior vena cava syndrome

A 42-year-old woman with a past medical history of multiple sclerosis (MS), recurrent deep vein thromboses (DVTs) of the lower extremities, and pulmonary embolism (PE) was hastily sent to the emergency department (ED) due to acute worsening upper body swelling. On initial presentation, she was anxious with mild tachypnea and hypoxemia requiring a low dose of supplemental oxygen. She also had worsening headache since admission. Her upper extremities and thorax were swollen and firm to palpation, in addition to facial plethora and neck edema (Figure 1). An indwelling catheter appeared to be positioned in the right subclavian vein without obvious edema, erythema, or tenderness. The patient reported the catheter was placed months ago for the intermittent infusion of natalizumab to treat MS. She had since been transitioned to an alternative subcutaneous medication, yet she continued to retain the indwelling catheter. The patient endorsed a history of multiple provoked venous thromboembolism (VTE) in the past, either post-surgical or central catheter related; however, she admitted non-compliance with her anticoagulation. She also has a remote history of intravenous (IV) drug use but states she is not currently using any recreational drugs.

**Figure 2:**
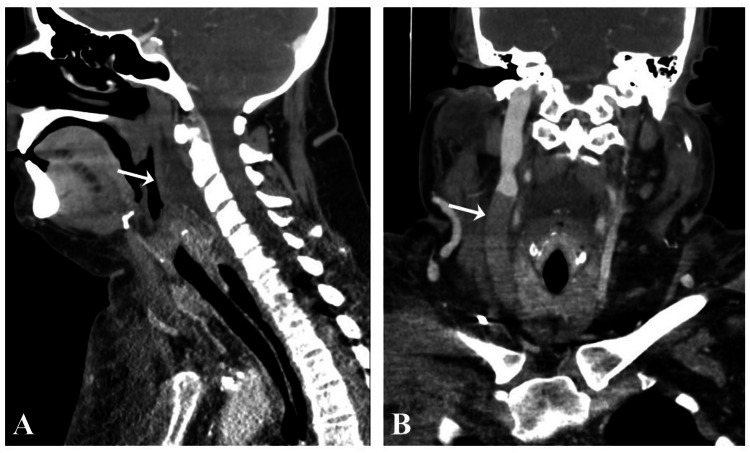
Contrast-enhanced CT scan of the neck revealing (A) retropharyngeal soft tissue edema and/or effusion with retropharyngeal/prevertebral soft tissue thickening (mild generalized subcutaneous soft tissue edema of the visualized bilateral anterior chest wall, neck, and submandibular regions) and (B) diffuse hypo-enhancing appearance of the lower portion of the right internal jugular vein. CT: computed tomography

A computed tomography (CT) scan of the soft tissues in the neck showed retropharyngeal soft tissue edema (Figure 2A) and diffuse hypo-enhancing appearance of the right internal jugular vein (Figure 2B).

**Figure 3:**
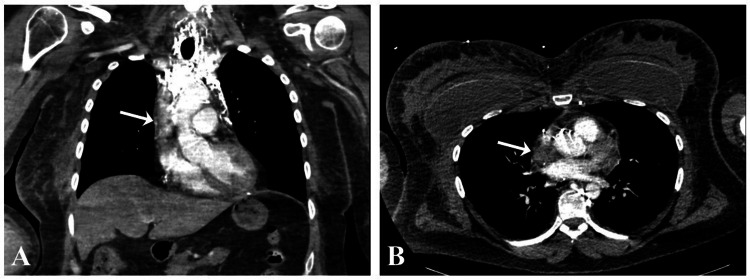
CT angiography of the chest showing SVC stenosis/high-grade partial obstruction with interval visualization of the numerous mediastinal venous collaterals in (A) coronal view and (B) axial view. CT: computed tomography; SVC: superior vena cava

Subsequent CT angiography of the chest suggested high-grade partial obstruction of the SVC (Figure 3); hence, a diagnosis of SVCS secondary to catheter-associated thrombosis was established. Continuous heparin infusion was initiated in parallel to emergent consultation with interventional radiology and vascular surgery. A transthoracic echocardiogram was negative for cardiological complications secondary to the acute extensive VTE.

**Figure 4:**
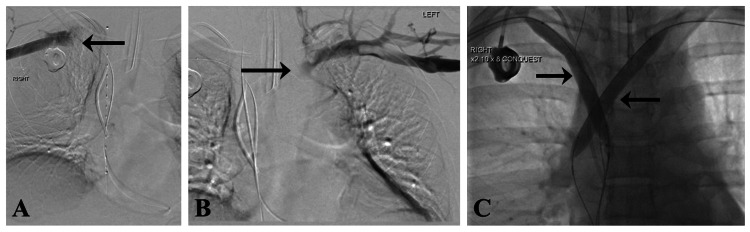
Bilateral venogram suggesting persistent occlusive thrombus despite overnight thrombolysis and percutaneous transluminal balloon venoplasty. (A) Right subclavian venogram showed the thrombus in the right subclavian vein, right brachiocephalic vein, and SVC (black arrow shows the occlusion). (B) Left brachiocephalic venogram (black arrow shows the occlusion). (C) Percutaneous transluminal venoplasty of the SVC and bilateral brachiocephalic and subclavian veins (black arrows show the balloons). SVC: superior vena cava

A central venogram (Figure 4A-4B) revealed occlusive thrombi in the SVC, right brachiocephalic vein, subclavian vein, and internal jugular vein. Consequently, ultrasound (US)-assisted infusion catheters for thrombolysis were placed for 24 hours yet were unsuccessful; balloon venoplasty (Figure 4C) also failed to reliably resolve the occlusion.

**Figure 5:**
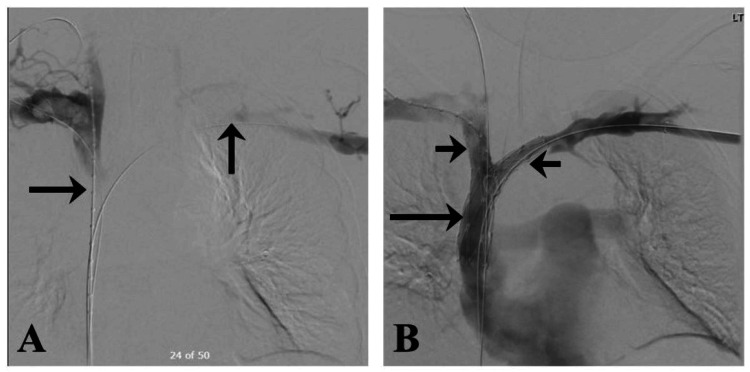
Completion venography revealing successful revascularization. (A) Superior vena cavogram before endovascular revascularization (black arrows show the occlusion). (B) Venogram after the endovascular revascularization of the SVC and bilateral innominate with stent shows widely patent SVC (long black arrow) and innominate veins (short black arrows). SVC: superior vena cava

The subsequent endovascular revascularization of the SVC with stenting (Figure 5) and the removal of her indwelling catheter finally led to resolution and improvement in the patient's symptoms and signs. The patient resumed rivaroxaban for the long-term management of VTEs. Strict adherence to the anticoagulation was re-enforced. The patient was advised to have a hypercoagulability evaluation with a hematologist after discharge.

## Discussion

Central IV catheter was first brought to the public's attention by Dr. Werner Forssmann, a pioneer of cardiology who successfully inserted a ureteral catheter through his left antecubital vein into his right atrium [3]. Since then, this technique has been broadly used in clinical practice for total parenteral nutrition administration, dialysis, plasmapheresis, medication administration, and hemodynamic monitoring and to facilitate further complex interventions such as transvenous pacemaker placement [4]. Unfortunately, it can also lead to numerous short-term and long-term complications, including catheter-related thrombosis (CRT) [5]. CRT is estimated to contribute to 10% of all DVTs in adults [6], which could lead to significant medical complications in addition to increasing healthcare spending.

The majority of catheter-related venous thrombosis occurs in the upper extremities. Clinical manifestations are variable, and the severity of clinical symptoms is positively correlated to the acuity and extent of the venous obstruction whereas inversely correlated to the development of the venous collateral circuits. Common presenting symptoms include swelling of the upper extremities, face, or neck; head fullness which is typically exacerbated by bending forward or lying down; and respiratory distress and swallowing issues due to soft tissue edema-related oropharyngeal lumen narrowing [1]. Worrisome signs include stridor, as this is usually indicative of laryngeal edema, as well as confusion and obtundation, since these may indicate cerebral edema. Although SVCS is now known not to be a major threat to life in most clinical scenarios, evidence of respiratory and neurologic compromise can be associated with serious or fatal outcomes [7].

Upon the diagnosis of SVCS, elevating the patient's head as a simple maneuver should be initiated to decrease venous pressure, and further management depends on the underlying etiology of SVCS. For catheter-related SVCS, removal of the thrombogenic stimulus should be considered along with anticoagulation therapy and catheter-directed thrombolysis [8]. Endovascular therapy, which can provide immediate symptom relief, is now widely considered the first-line treatment for SVCS [9]. Venoplasty and stent placement can be reserved for acute or subacute thrombus cases that failed catheter-based thrombolysis or thrombectomy, as presented in our case.

The favorable clinical outcome with our patient should be attributed to the effective multidisciplinary teamwork leading to the rapid diagnosis and appropriate management of a life-threatening medical emergency. Besides the acute management of SVCS, re-assessment of the indication for central venous devices and patients' underlying risk factors for thrombosis is equally important in catheter-related SVCS cases. In our case, the clinical decision-making for subcutaneous central venous port placement was not routine. Implantable ports or subcutaneous venous access devices (SVADs) are most commonly associated with chemotherapy infusion where the infusate acts as an irritant on peripheral vasculature, justifying the hemodilutory effect of central access. However, Walser [10] takes the position that patients treated in the outpatient setting with any intermittent, chronic infusion therapy with poor peripheral access as a result of repeated vascular trauma are ideal candidates for SVAD placement. Additionally, the expert panelists responsible for the formulation of the Michigan Appropriateness Guide for Intravenous Catheters (MAGIC) deem appropriate the use of ports with a greater than 31-day period of therapy in patients with peripherally compatible infusate (like monoclonal therapy) or difficult venous access [11]. The patient's monoclonal therapy for MS was performed over the span of several years, so it seems plausible that port placement was relatively indicated. This type of therapy is an unusual application for SVAD and, arguably, might have been forgone for more conservative venous routes.

Despite these relative indications, the patient's history of non-adherence to thrombolytic therapy, consistent successful peripheral access, and distant history of IV drug use all represent significant relative contraindications to the decision for SVAD placement. The patient's history of VTE and resultant pulmonary embolisms which were precipitated by non-adherence to thrombolytic therapy ought to have brought significant hesitation to clinical decision-making, especially considering the intrinsically prothrombotic nature of ports [12]. Also, consistent utilization of peripheral venous access for phlebotomy, drug and contrast administration, and fluid resuscitation documented over the course of years since port placement naturally questions the relative indication of access difficulties. Finally, clinical decision-making ought to recognize the relative and concerning history of IV drug use for the placement of central access devices.

Even with this discussion concerning the initial placement of the patient's port, multiple clinical concerns reasonably ought to have necessitated the prompt removal of the device. Our patient demonstrated increased utilization of emergency services at multiple area hospitals. At each of these encounters, the history of infusion therapy cessation was consistently documented. However, much of the documentation did not identify the clinician responsible for the maintenance of the port; therefore, the clinical concern of an idle and unmaintained SVAD was not documented. This medical error represents a significant opportunity; wider knowledge of the indications for SVADs and consistent monitoring could have decreased the iatrogenic burden in our patient.

This case highlights the necessity of scrutinizing the indication of chronic IV catheterization and continually evaluating the possibility of its removal if the underlying indication is no longer relevant. Assessment of underlying risk factors for thrombosis is essential to ensure appropriate patient selection. In this situation, the suspected hypercoagulability and the patient's concurrent non-adherence to anticoagulation treatment, if previously known, should have provided sufficient justification to avoid the use of a long-term indwelling venous catheter.

## Conclusions

The usage of chronic IV devices has become one of the most common etiologies for benign conditions leading to SVCS, which can be a life-threatening medical emergency. In our case, some of these IV devices are not indispensable, or other treatment options may exist. This case highlights the necessity of scrutinizing the indication of chronic IV catheterization and continually evaluating the possibility of its removal if the underlying indication is no longer relevant, in order to minimize chronic indwelling catheter-related complications.
